# Microecologics and Exercise: Targeting the Microbiota–Gut–Brain Axis for Central Nervous System Disease Intervention

**DOI:** 10.3390/nu17111769

**Published:** 2025-05-23

**Authors:** Zhixing Peng, Tingting Hou, Keer Yang, Jiangyu Zhang, Yu-Heng Mao, Xiaohui Hou

**Affiliations:** 1 School of Exercise and Health, Guangzhou Sport University, Guangzhou 510500, China; 17798442576@163.com (Z.P.); 17852776927@163.com (T.H.); keryang014@126.com (K.Y.); 18933517976@163.com (J.Z.); 2 Guangdong Key Laboratory of Human Sports Performance Science, Guangzhou Sport University, Guangzhou 510500, China

**Keywords:** microecologics, sport, neurological disorders, autism, microbiota–gut–brain axis, probiotics, prebiotics, synbiotics

## Abstract

The gut microbiota (GM) may play a crucial role in the development and progression of central nervous system (CNS) diseases. Microecologics and exercise can influence the composition and function of GM, thereby exerting positive effects on the CNS. Combined interventions of exercise and microecologics are expected to more comprehensively and effectively address CNS diseases through the microbiota–gut–brain axis (MGBA), potentially outperforming single interventions. However, there is currently a lack of relevant reviews on this topic. In this review, we examine the associations between changes in the microbiota and CNS diseases, including Alzheimer’s disease (AD), Parkinson’s disease (PD), multiple sclerosis (MS), and autism spectrum disorder (ASD). We also summarize studies on various types of microecologics (such as probiotics, prebiotics, synbiotics, and postbiotics) and exercise in improving CNS disease symptoms. Although current individual studies on microecologics and exercise have achieved certain results, the mechanisms underlying their synergistic effects remain unclear. This review aims to explore the theoretical basis, potential mechanisms, and clinical application prospects of combined interventions of microecologics and exercise in improving CNS diseases through the MGBA, providing a scientific basis for the development of more comprehensive and effective therapeutic interventions.

## 1. Introduction

In recent years, with the intensification of population aging and the alteration of environmental factors, the incidence of various central nervous system (CNS) diseases has risen significantly, posing one of the major challenges faced globally [1]. CNS diseases mainly include Alzheimer’s disease (AD), Parkinson’s disease (PD), multiple sclerosis (MS), and autism spectrum disorder (ASD), etc. [2,3]. At present, such diseases not only lead to severe cognitive and motor function impairment of patients but also cause heavy burdens to patients’ families and society due to their high disability rate and lack of effective treatment [4,5]. Therefore, it is important to find effective measures. Traditional treatment methods have typically focused on single-drug interventions or physical rehabilitation. Although they can relieve symptoms to a certain extent, there are still many limitations in the comprehensive prevention and treatment of diseases. For example, Parkinson’s disease drugs (such as levodopa) can improve motor function, but long-term use leads to reduced efficacy and side effects [6]. Physical therapies (such as acupuncture and manual therapy) can regulate neural functions and reduce inflammation, but their repair effects on severe neurodegenerative lesions are limited. They rely on individualized plans and have unstable curative effect [7]. Compared with drug treatment and physical rehabilitation, microecologics (microecologics are a very important class of supplements, which mainly contain normal microorganisms that have a positive effect on the host and the metabolites of these microorganisms, or substances that can promote the growth of normal microorganisms, i.e., probiotics, prebiotics, synbiotics, and postbiotics) [8] and exercise [9] have attracted increasing attention as two potential intervention strategies. This is mainly because they have the potential to regulate the gut microbiota (GM) and improve the outcomes of CNS diseases. Importantly, these interventions are characterized by high safety and low cost [10,11]. Single microecologics or exercise have been shown to alleviate CNS diseases [12,13,14]. However, some studies have found that the combination of the two can improve CNS diseases more significantly. For instance, it was found that for AD mice, the combined application of microecologics and exercise was more effective than an individual intervention [15]. The purpose of this review is to summarize the current evidence on the single and combined strategies of these two interventions in the context of CNS diseases. In addition, it aims to highlight the great potential of comprehensive approaches to address CNS diseases, particularly through the microbiota–gut–brain axis (MGBA).

## 2. Central Nervous System Diseases and Microbiome–Gut–Brain Axis

### 2.1. Central Nervous System Diseases

CNS diseases collectively refer to a class of disorders that affect the structure or function of the brain and spinal cord. The pathological processes may include abnormalities in neurons, glial cells, vascular networks, or supporting tissues, leading to impairments in sensory, motor, cognitive, or autonomic regulatory functions [16]. As shown in Table 1, an increasing number of studies have revealed a close association between microbial dysbiosis and the development and progression of various neurodevelopmental and neurodegenerative diseases [17]. A large number of review articles have comprehensively and thoroughly addressed this issue. Therefore, this section will only briefly introduce these associations [16,18,19].

#### 2.1.1. Alzheimer’s Disease

AD is a prevalent neurodegenerative disorder characterized by progressive cognitive dysfunction and is one of the most common causes of dementia in the elderly. It is estimated that approximately 55 million people worldwide are affected by AD [39]. Its pathogenesis is closely associated with the abnormal generation and accumulation of amyloid-beta (Aβ) peptides [40,41]. Aβ, specifically the Aβ 42 peptide, composed of 40–42 amino acids, is produced through the proteolytic cleavage of amyloid precursor protein (APP). In the pathogenesis of AD, Aβ triggers a series of complex neuroinflammatory responses, exerting profound effects on the structure and function of neuronal cells [42].

Emerging research has demonstrated complex and intimate interactions between the GM and microglia in AD [43]. For instance, in a triple-transgenic AD (3xTg-AD) mouse model, many key pathological features of AD, such as Aβ plaque formation, tau protein hyperphosphorylation, synaptic dysfunction, and microglial activation, appear to be influenced by the GM [39]. Comparisons between germ-free (GF) and specific pathogen-free (SPF) 3xTg-AD mice have revealed that pathological AD conditions in SPF 3xTg-AD mice are significantly more severe. Notably, fecal microbiota transplantation (FMT) of GM from AD patients into GF 3xTg-AD mice restored key AD pathological features and induced microglial activation [44].

Furthermore, studies have indicated that certain pathogenic bacteria may exacerbate AD. For example, one study demonstrated that *Helicobacter pylori* can induce tau protein hyperphosphorylation and promote the secretion of inflammatory mediators and amyloid proteins. Encouragingly, subsequent treatment of *H. pylori* infection using triple eradication therapy has been shown to improve cognitive parameters in AD patients. These findings suggest a tight association between GM-related factors and AD, providing new insights and potential intervention targets for the prevention and treatment of AD in the future [45].

#### 2.1.2. Parkinson’s Disease

PD is a common central nervous system disorder characterized primarily by tremors, muscle rigidity, gait abnormalities, and bradykinesia. The condition progresses over time, gradually worsening and causing significant inconvenience and distress in patients’ daily lives [46]. The primary pathological hallmark of PD is the loss of dopaminergic neurons in the substantia nigra, accompanied by the accumulation of α-synuclein and the deposition of Lewy bodies in the remaining neurons [47]. Emerging evidence suggests that α-synucleinopathy may initiate in the enteric nervous system (ENS) and propagate to the CNS during early PD [48,49]. Consequently, gastrointestinal symptoms and alterations in the GM are common phenomena in the course of PD. However, the underlying mechanisms linking the GM to PD have only been gradually uncovered in recent years [50]. For instance, studies by Sampson et al. have demonstrated that in α-synuclein-overexpressing (ASO) mice, the development of α-synuclein pathology, microglial activation, and motor deficits appears to be influenced by the GM. Specifically, when the GM from PD patients was transferred into GF ASO mice via FMT, the mice exhibited a restoration of key disease characteristics that were originally absent, including motor dysfunction mediated by α-synuclein. This finding directly highlights the critical role of the GM in the pathogenesis of PD-related pathology [51].

Furthermore, research has shown that FMT from mice treated with 1-methyl-4-phenyl-1,2,3,6-tetrahydropyridine (MPTP) induces motor dysfunction and neurotransmitter loss in healthy mice [52]. In contrast, transferring GM from healthy mice to MPTP-induced mice via FMT has been observed to ameliorate gut dysbiosis and PD-related pathological features, such as intestinal inflammation, neuroglial activation, neurotransmitter abnormalities, and motor dysfunction. These findings from different perspectives corroborate the pivotal role of the gut–brain axis (GBA) in the pathogenesis of PD [53]. Additionally, studies in non-human primate models (baboons) have provided further insights. Injection of patient-derived α-synuclein aggregates into the gut or striatum of these animals has been shown to induce nigrostriatal lesions and ENS pathology, further elucidating the role of the GBA in the propagation of PD [2].

Notably, certain gut bacteria have been validated as highly sensitive biomarkers for PD diagnosis. For example, *Prevotellaceae* has been found to exhibit a sharp decline in abundance following the onset of PD, while an increase in the abundance of *Enterobacteriaceae* has been positively correlated with the severity of PD symptoms. Particularly significant is a recent diagnostic model combining *Prevotellaceae* abundance and constipation status, which demonstrated high diagnostic efficacy with a specificity of 90.3%. This model offers a promising avenue for the early diagnosis of PD [24].

#### 2.1.3. Autism Spectrum Disorder

ASD is an early-onset neurodevelopmental disorder (NDD) characterized by deficits in social cognition, communication impairments, and the presence of restricted, repetitive behaviors (American Psychiatric Association, 2013) [54,55]. The etiology of ASD has long been a focal topic of interest in medical research [56]. Due to the high heterogeneity of ASD, significant variations exist among patients in terms of symptom presentation, severity, and other aspects, leaving its underlying pathogenic mechanisms still unclear [57].

A meta-analysis revealed that children with ASD are significantly more likely to experience gastrointestinal (GI) symptoms compared to their neurotypical peers. Furthermore, the presence of GI disorders in these children tends to exacerbate their existing neurobehavioral symptoms [58]. Research has also highlighted substantial differences in GM composition between individuals with ASD and neurotypical individuals. For instance, beneficial bacterial genera such as *Bifidobacterium* show a marked reduction in abundance in the guts of individuals with ASD, while potentially pathogenic genera like *Clostridium* exhibit increased abundance [59].

Another study demonstrated that levels of beneficial bacterial groups, such as *Prevotella* and *Coprococcus*, are significantly lower in the guts of children with ASD compared to healthy controls [60]. Animal studies have shown similar changes in the GM of valproic acid-induced autistic rat models [61]. Moreover, adults with ASD exhibit significantly elevated levels of bacterial lipopolysaccharides (LPS) and inflammatory cytokines, such as IL-1β and IL-6, in their serum compared to healthy controls [62]. These findings underscore the role of the microbiome in ASD.

Furthermore, FMT has been employed in treating ASD patients. Transferring GM from healthy individuals to ASD patients has led to moderate improvements in both GI comorbidities, such as constipation and abdominal pain, and behavioral symptoms, including repetitive behaviors and social skill deficits [63]. FMT has achieved satisfactory results in preventing the recurrence of Clostridioides difficile infection, but these positive outcomes have only been partially replicated in other diseases [64]. Simultaneously, multiple preclinical and clinical studies have demonstrated significant alterations in the fecal or gut luminal metabolites of ASD patients. These changes are likely driven by GM dysbiosis. Certain microbiota-derived metabolites, such as short-chain fatty acids (SCFAs), neurotransmitters, and various amino acids, have been shown to exert considerable effects on CNS development and ASD-related phenotypes in animal models [29].

The GM can influence neurodevelopmental processes in ASD through various pathways, including metabolite production, immune modulation, and neural signaling. These pathways often act concurrently and are intricately interconnected, collectively regulating the progression of ASD. For specific subtypes of ASD or particular symbiotic bacteria, there may exist a dominant primary pathway that mediates the microbiota’s regulatory effects on ASD. However, further in-depth and detailed research is required to identify and clarify these mechanisms [65].

#### 2.1.4. Multiple Sclerosis

MS is a CNS and inflammatory disease that affects more than 2 million people worldwide. It is characterized by immune-mediated demyelination of neuronal axons. The loss of myelin can lead to various neurological disorders of varying severity, including motor, sensory, visual, autonomic, and cognitive impairments [66,67,68]. An imbalance in CD4^+^ T-cell subsets (overactivation of Th1/Th17) drives the production of pro-inflammatory cytokines, recruiting immune cells to infiltrate the CNS and mediating myelin-specific immune attacks [69,70]. Defective immunosuppressive functions of regulatory T cells (Tregs) in MS patients may further exacerbate autoimmune responses [71,72]. The pathogenesis of MS is thought to originate from the immune system and is significantly influenced by both genetic and environmental factors [73]. Given that the GM simultaneously regulates innate immune signaling and certain physiological processes in the CNS, it has been hypothesized to be associated with the pathogenesis of MS [74].

The experimental autoimmune encephalomyelitis (EAE) model, a multifocal demyelinating disease animal model mediated by CD4^+^ T cells (particularly Th1/Th17 subsets), is widely used to simulate MS [75]. Studies have found that oral administration of antibiotics can inhibit neuroinflammation by enhancing the recruitment and proliferation of FoxP3^+^ Tregs, significantly reducing the severity of EAE [76]. A recent study found that segmented filamentous bacteria (SFB), a key pathogenic factor, can specifically induce Th17 cell differentiation and exacerbate EAE pathology [76,77]. Conversely, introducing SFB into GF mice restored Th17 cell levels in the CNS and improved motor function, suggesting that the GM maintains Th17 homeostasis through immunoregulatory mechanisms [77].

Additionally, the role of GM dysbiosis in promoting the development of MS has been discussed in MS patients. A clinical trial specifically investigating the GM characteristics of MS patients found that certain microbial taxa, such as *Akkermansia muciniphila* and *Acinetobacter calcoaceticus*, were elevated in these patients. Subsequent transplantation of these bacteria from MS patients into GF mice resulted in enhanced pro-inflammatory T-cell responses and reduced Treg activity, leading to the exacerbation of EAE [78].

GM alterations are closely related to the occurrence and development of MS, which has been further confirmed and strengthened in other related studies. For example, elevated levels of *Fusobacterium* have been linked to an increased likelihood of MS relapses. These findings implicate Fusobacterium dysbiosis as a potential biomarker for MS relapse risk, warranting further validation in longitudinal studies [79]. Furthermore, FMT experiments have demonstrated that mice receiving fecal microbiota from MS patients exhibited worse EAE phenotypes and fewer anti-inflammatory regulatory T cells compared to those receiving fecal microbiota from healthy individuals. This provides more direct evidence of the close association between the GM and MS, as well as its significant impact on disease progression. Collectively, these findings lay the foundation for future research on the microbiota and pathways involved in the progression of MS. They also provide valuable insights and a strong basis for further exploration of the pathogenesis of this disease and the identification of effective therapeutic targets [78].

In addition to the four diseases mentioned above, CNS disorders such as amyotrophic lateral sclerosis (ALS), Huntington’s disease (HD), and attention deficit hyperactivity disorder (ADHD) also appear to be closely associated with MGBA dysregulation [80,81,82]. However, due to the relatively low incidence of these diseases, they are not discussed in this section or were within the scope of this study.

### 2.2. Microbiota–Gut–Brain Axis

**Figure 1:**
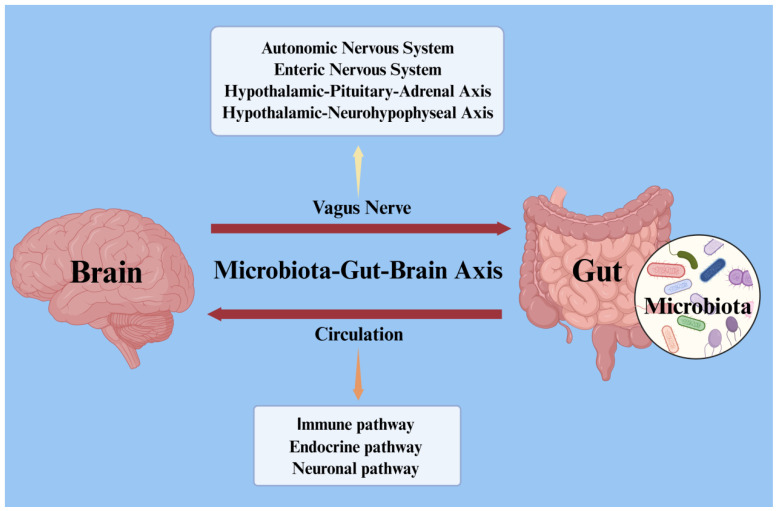
The microbiota–gut–brain axis. The bidirectional communication between the brain and the gut microbiota is mediated by multiple pathways, including immune pathways, endocrine pathways, neuronal pathways, autonomic nervous system (ANS), enteric nervous system (ENS), hypothalamic–pituitary–adrenal (HPA) axis, hypothalamic–neurohypophyseal (HN) axis, and vagus nerve.

The gut and brain are intricately connected, with the resident microbial communities playing a pivotal regulatory role. These communities, which include symbiotic bacteria, fungi, viruses, and archaea (archaea are microorganisms that can colonize extremely inhospitable environments, with stable proteins and enzymes enabling survival and function under extreme conditions [83]), are collectively referred to as the microbiome [84]. They represent the highest density and absolute abundance of microorganisms within the human body. The CNS acts as a “control center”, regulating various physiological processes in the gut, such as GI motility, secretion, and digestive functions. In turn, the GM exerts influence on brain function through multiple dimensions, including neural transmission, humoral signaling, and immune regulation [85]. As shown in Figure 1, further exploration of the bidirectional communication pathways between the brain and the GM reveals that this interaction is mediated by various mechanisms, including the immune system, neuroendocrine system, ENS, circulatory system, and vagus nerve. These pathways are collectively termed the MGBA [86]. The MGBA primarily relies on three key pathways: immune, endocrine, and neuronal. Importantly, these pathways do not function in isolation, but interact and are tightly interconnected [87,88].

#### 2.2.1. Immune Pathways

The GM can significantly influence the activity and function of the immune system [89]. Specifically, the GM interacts with the immune system through its own components and microbial metabolites, such as SCFAs, secondary bile acids, trimethylamine *N*-oxide (TMAO), and other bioactive molecules. Together, they regulate local immunity within the gut and influence the CNS through the circulatory system [18,90,91,92]. The GM and its metabolites must penetrate the gut barrier to enter the systemic circulation. Therefore, the gut barrier plays a critical role in immune pathways [93,94].

For example, SCFAs, one of the most extensively studied microbe-derived metabolites, are saturated fatty acids with fewer than six carbon atoms. The major SCFAs found in humans are acetate (C2), propionate (C3), and butyrate (C4), which account for 95% of the total SCFA repertoire [95]. SCFAs have been shown to actively participate in strengthening gut barrier function by enhancing tight junctions between intestinal epithelial cells and promoting the repair and maintenance of the intestinal mucosal layer. This significantly improves the integrity of the gut barrier, reduces gut permeability, and effectively prevents the invasion of harmful microbes. By doing so, SCFAs reduce the risk of intestinal infections and systemic inflammatory responses [54]. SCFAs can also influence adaptive immune responses by directly or indirectly affecting the maturation and activation of T cells, macrophages, dendritic cells (DCs), and neutrophils [96,97]. Neutrophils, the most common type of granulocyte produced in the bone marrow, are a crucial component of the innate immune system and are among the first responders at sites of inflammation [98]. SCFAs may directly impact neutrophils by inhibiting histone deacetylases (HDACs), thereby regulating the production of pro-inflammatory cytokines such as tumor necrosis factor (TNF). This in turn influences peripheral immune system activity and subsequently modulates brain function [99].

The GM is also essential for the development and activation of innate immune cells in the brain [100]. Cytokines, small protein molecules secreted by immune cells (such as macrophages and T cells), and intestinal epithelial cells mediate bidirectional communication between the gut and the CNS through the regulation of inflammatory responses and immune homeostasis within the GBA [101]. The GM communicates with the brain via the systemic immune system and circulating cytokines [18]. Once activated, gut immune cells (such as macrophages and dendritic cells) release cytokines into the systemic circulation, which can cross the blood–brain barrier (BBB) and influence the immune microenvironment of the CNS [54].

The BBB is a critical barrier that separates the brain’s microenvironment from the rest of the body, and is composed of tight junction proteins that connect mural cells and microvascular endothelial cells [18]. The BBB plays a vital role in protecting the brain from pathogens and harmful immune responses that could damage neurons and their connections [102]. The permeability of the BBB is influenced by the GM, as studies have shown that compared to control mice, GF mice exhibit increased BBB permeability, partly due to reduced expression of tight junction proteins such as occludin and claudin 5 [103]. The increased permeability of BBB may exacerbate neuroinflammation by amplifying cytokine cascades and immune cell activation [104].

#### 2.2.2. Endocrine Pathway

The GM can regulate the CNS by secreting a variety of metabolites and synthesizing neurotransmitters and neuromodulators [90,105]. These metabolites or products include gamma-aminobutyric acid (GABA), serotonin, dopamine, norepinephrine, acetylcholine, histamine, secondary bile acids, sulfated 4-ethylphenylsulfate (4-EPS), SCFAs, and others [18,90,91]. As one of the most important products of GM metabolic activity, SCFAs are actively involved in regulating the functions of the GBA, demonstrating their unique biological activities. In the colon, SCFAs can activate G protein-coupled receptors (GPCRs), significantly enhancing the release of hormones such as glucagon-like peptide 1 (GLP-1) from enteroendocrine L cells. These gut hormones carry information from the gut and transmit signals throughout the body via the circulatory system, particularly exerting profound effects on brain function. They possess the ability to regulate mood, improve memory, and enhance learning, thus playing a critical role in the communication and functional regulation of the GBA [106,107,108].

Notably, SCFAs also possess a unique mode of information transmission. By interacting with free fatty acid receptors (FFARs), they can directly activate vagal afferent nerves, thereby rapidly and precisely signaling to the brain and establishing a fast neural communication pathway between the gut and the brain [99]. For example, GLP-1 has been shown to significantly enhance memory and learning abilities in mice [109], promote hippocampal neuroplasticity, and exert neuroprotective effects [110,111]. In AD mouse models, GLP-1 effectively reduces the formation of amyloid-beta plaques and inhibits the overactivation of microglia, thereby slowing disease progression and improving the pathological state of the brain [110].

Additionally, the hypothalamic–neurohypophyseal (HN) axis and the hypothalamic–pituitary–adrenal (HPA) axis play important roles in the endocrine pathway. The HN axis is an important component of the neuroendocrine system, primarily responsible for regulating the secretion of oxytocin (OXT) and arginine vasopressin (AVP) [112,113]. The HPA axis regulates the stress response, metabolism, and immune function through the coordinated action of the hypothalamus, the pituitary gland, and the adrenal glands [114,115]. The HN axis and HPA axis communicate with the gut through endocrine signals (hormones) and neural pathways (vagus nerve). For instance, oxytocin, a key neuropeptide in the HN axis, precisely regulates interactions within the nucleus accumbens serotonergic system, stabilizing neurotransmitter transmission and regulation. It also finely tunes the limbic activity of the amygdala, playing a crucial role in shaping and regulating social behavior [18,105,116].

Along the HPA axis, a critical endocrine regulatory pathway, peripheral cortisol dysregulation has been implicated in ASD pathophysiology, suggesting that HPA axis dysregulation may contribute to the pathogenesis of ASD. Further research has revealed that specific bacterial species, such as *Enterococcus faecalis*, can effectively inhibit the increase in glucocorticoid levels following social stress and significantly promote social behavior in mice. This indicates a connection between the GM and the HPA axis, suggesting that the GM can influence the HPA axis through certain mechanisms, thereby impacting the development and progression of neuropsychiatric disorders [117,118].

#### 2.2.3. Neuronal Pathway

The neuronal pathways underlying bidirectional gut–brain communication are relatively straightforward. The GI tract is unique among visceral organs in that it possesses its own intrinsic nervous system—the ENS [119,120]. At the same time, the extrinsic nervous system plays a critical role in gastrointestinal physiology, with the vagus nerve being particularly prominent among the various neural networks. As the tenth cranial nerve, the vagus nerve is uniquely structured, originating in the brainstem and extending to innervate the GI tract and ENS [121]. The vagus nerve represents the most direct and well-studied connection between the gut and the CNS [122]. In terms of distribution, nearly the entire digestive system is under the control of the vagus nerve, with sensory/afferent fibers accounting for approximately 80% and motor/efferent fibers for around 20%. Sensory fibers originate from neurons in the nodose ganglion, while motor fibers arise from neurons in the dorsal motor nucleus of the vagus (DMV) [123].

Notably, the vagus nerve afferent pathway serves as a critical route for chemical signal transduction and plays an essential role in the bidirectional exchange of information between the gut and the CNS [54]. The afferent nerve endings of the vagus nerve innervate multiple layers of the digestive wall, with mucosal afferents terminating in the lamina propria of the intestinal mucosa [123]. Receptors on the vagus nerve are highly sensitive and capable of detecting the presence of various substances, including inflammatory chemicals, dietary components, and bacterial metabolites, transmitting corresponding signals to the CNS and thus establishing a bridge for information transfer between the gut and the brain [124].

Emerging evidence suggests that vagotomy disrupts the normal regulation of the brain by microbiota-derived metabolites or host-secreted peptides or neurotransmitters triggered by microbes, such as serotonin, oxytocin [116], GABA [125], and brain-derived neurotrophic factor (BDNF) [126]. For example, *Lactobacillus rhamnosus* JB-1 has a direct impact on neurotransmitter receptors, inducing region-dependent changes in GABAB1b mRNA expression in the brain. This process is primarily regulated by the vagus nerve, as demonstrated by studies showing that mice subjected to bilateral subdiaphragmatic vagotomy are unable to achieve this effect [54]. In addition to the vagus nerve, the ENS also plays a critical role as an important mediator of the MGBA. Animal studies and cross-sectional research have revealed a strong association between the ENS and CNS diseases, particularly in conditions such as ASD, which often co-occur with gastrointestinal comorbidities [127].

Furthermore, studies have found that gut bacteria can directly activate neurons. Toll-like receptors (TLRs) 3 and 7, which detect viral RNA, and TLRs 2 and 4, which detect peptidoglycan and lipopolysaccharides, are present in the gut nervous systems of both mice and humans [86,128]. Polysaccharide A (PSA) from *Bacteroides fragilis*, as well as isolated polysaccharides from *Lactobacillus rhamnosus* JB-1 and *Bacillus fragilis*, have been shown to stimulate gut afferent neurons in vitro [129]. Moreover, the GM has the ability to directly produce neuroactive molecules, such as 5-aminovaleric acid (5-AVA), taurine, and sulfated 4-EPS. These molecules exert diverse effects on neurodevelopmental processes, including myelination and oligodendrocyte maturation [54].

Although it is known that gut microbes and their metabolites, such as SCFAs, may affect CNS diseases through vagal, immune, or endocrine pathways [85,87,88], the precise molecular mechanisms and signaling pathways remain incompletely understood. In addition, most of the existing studies are based on animal models. Although the GF mouse model is helpful to study the effect of microflora depletion, its immune and nervous system development are significantly different from those of humans, and it cannot completely simulate the human pathological process [130]. In addition, while correlative analyses (e.g., microbiota abundance vs. disease phenotype) dominate current studies, causal relationships require rigorous validation through genetic knockout or metabolite intervention experiments. In the future, more studies are needed to reveal the association between the MGBA and CNS diseases and promote clinical application.

## 3. The Impact of Microecologics on Central Nervous System Diseases

### 3.1. Classification and Functions of Microecologics

Microecologics are a very important class of supplements and mainly contain normal microorganisms that have a positive effect on the host and the metabolites of these microorganisms or substances that can promote the growth of normal microorganisms. This category encompasses probiotics, prebiotics, synbiotics (i.e., both probiotics and prebiotics), and postbiotics. They exert their effects by secreting specific enzymes, stimulating the growth of beneficial bacteria, inhibiting pathogen adhesion/colonization, and maintaining biological barriers, thereby regulating and preserving microbial homeostasis to enhance host health [8,131], as shown in Table 2.

#### 3.1.1. Probiotics

The term “probiotics” first appeared in 1974. The World Health Organization (WHO) defined it as live microorganisms that have a beneficial effect on the host’s health when consumed in appropriate amounts [146]. Some commonly used probiotic microorganisms include specific strains of *Lactobacillus rhamnosus*, *Lactobacillus reuteri*, *Bifidobacterium* species, *Lactobacillus casei* strains, the *Lactobacillus acidophilus* group, *Bacillus coagulans*, *Escherichia coli* strain Nissle 1917, certain *Enterococcus* species, especially *Enterococcus faecium* SF68, and yeast *Saccharomyces boulardii*, etc. These probiotics can be applied either in single form or in combination. The components of probiotic products are diverse, which may be a single strain or a mixture of two or more strains. The most common beneficial effects of probiotics are reflected in the restoration of the GM and improvement in gut and immune homeostasis [147,148].

In addition, an increasing number of studies have shown that probiotics can regulate central nervous system diseases, including normalizing anxiety and depression-like behaviors [125,149], and alleviating ASD-related symptoms to a certain extent [150]. For example, a randomized, double-blind, placebo-controlled study reported that compared with a placebo group, consuming *Lactobacillus plantarum* PS128 for 4 consecutive weeks significantly improved ASD-related symptoms [132]. In another double-blind, placebo-controlled, crossover study, after a 3-week *Lactobacillus plantarum* supplementation intervention, the GM of autistic children underwent significant changes, and some of their gastrointestinal symptoms were significantly improved. More importantly, the severity of autism-related behaviors, such as social impairment and repetitive stereotyped behaviors, was alleviated to a certain extent [151]. Probiotic treatment can also significantly improve the brain function of AD model mice [152]. Researchers selected a probiotic combination consisting of *Lactobacillus acidophilus*, *Bifidobacterium bifidum*, and *Bifidobacterium longum* to intervene in AD rats for 4 weeks. Compared with AD group rats that did not receive probiotics, the rats receiving probiotic intervention showed stronger cognitive abilities in spatial learning and memory, with more efficient neuronal signal transmission [133]. It is worth noting that in a randomized clinical trial of probiotic treatment involving 60 AD patients, Akbari et al. demonstrated that after taking *Lactobacillus* and *Bifidobacteria* through fermented milk for 12 weeks, the patients’ cognition was significantly improved [153].

Furthermore, the probiotic formulation SLAB51 has demonstrated multifaceted positive effects in studies on AD mice. Firstly, it can effectively alleviate cognitive impairment, Aβ aggregation, brain injury, and neuronal protein degradation alterations in AD mice [137]. Secondly, this preparation promotes antioxidant and neuroprotective effects by activating the SIRT1 pathway in the AD mouse model [154]. Notably, the latest in vitro experimental studies have shown that SLAB51 exhibits a unique neuroprotective mechanism in human neuroblastoma cells. Specifically, it can precisely regulate the BDNF pathway, significantly enhancing the PI3K/Akt, p-Trk, p-ERK5, and p-CREB pathways, while correspondingly inhibiting the p-JNK, ERK-1, and P75 pathways [155].

In a two-month daily dosing experiment on MS patients using a probiotic cocktail (a combination of *Lactobacillus paracasei*, *Lactobacillus plantarum*, *Lactobacillus acidophilus*, *Lactobacillus delbrueckii*, *Bifidobacterium longum*, *Bifidobacterium infantis*, *Bacillus subtilis*, and *Streptococcus thermophilus*), it was found that this probiotic combination can positively improve the symptoms of MS patients by regulating the GM structure and inducing an anti-inflammatory peripheral immune response [134]. In addition, in a randomized, double-blind, placebo-controlled trial, a probiotic group (Lactobacillus acidophilus, Bifidobacterium bifidum, Lactobacillus reuteri, and Lactobacillus fermentans) in PD patients improved biomarkers of inflammation, oxidative stress, and insulin metabolism after 12 weeks of treatment. Scores on the Movement Disorder Society Unified Parkinson’s Disease Rating Scale (MDS-UPDRS) in the intervention group decreased [136]. Although probiotic intervention has shown positive effects in CNS diseases, however, due to the heterogeneity, complexity, and dynamic changes in intestinal flora, many studies on intestinal flora have limitations. More experimental studies and high-quality large-sample clinical trials are needed in the future. The introduction of human-specific pathogenic genes can be considered in the future to optimize animal models and promote clinical translation effects.

#### 3.1.2. Prebiotics

Prebiotics were redefined in 2017 as “a substrate that is selectively utilized by host microorganisms and confers health benefits” [156]. Fibers, a major component of prebiotics, are indigestible food components that beneficially affect the host’s health by selectively stimulating the growth and/or activity of certain microbial genera in the colon [157]. There are various sources of prebiotics, such as breast milk, soybeans, inulin sources (such as artichokes, chicory roots, etc.), raw oats, unrefined wheat, unrefined barley, *yacón*, indigestible carbohydrates, and especially indigestible oligosaccharides [158]. Some of the most commonly studied prebiotics include inulin, fructo-oligosaccharide (FOS), lactulose, and galacto-oligosaccharide (GOS). The molecular structures and biological functions of these compounds have been widely studied.

Prebiotics play a significant role in enhancing brain function and preventing various neurological disorders. Specifically, they exhibit preventive effects against CNS diseases such as AD [159], dementia [160], PD [161], and ASD [162]. Among different prebiotics, studies on FOS are the most widely conducted. Studies have demonstrated that FOS exerts multiple beneficial effects on both the gut and brain. Firstly, FOS promotes the growth of beneficial bacteria while maintaining microbial diversity and stability. Secondly, FOS attenuates neuronal apoptosis and cerebral edema, improving neurotransmitter synthesis and release. Finally, FOS has been shown to ameliorate cognitive deficits and neurodegeneration in AD mice by modulating GM and activating the glucagon-like peptide-1 pathway [163]. In another study, administration of FOS derived from Morinda officinalis significantly improved learning and memory in AD rats induced by D-galactose and Aβ 1–42. These improvements were accompanied by reduced oxidative stress and inflammation, alongside elevated levels of key neurotransmitters, including 5-hydroxytryptamine (5-HT), 5-hydroxyindoleacetic acid (5-HIAA), acetylcholine, and dopamine. These benefits are likely attributed to FOS’s capacity to regulate gut–brain homeostasis [141].

In addition, other prebiotics have been shown to have positive effects on the CNS. For example, GOS interventions can ameliorate depressive and anxiety-like behaviors by targeting the MGBA, showing beneficial effects in chronically stressed mice [164]. In addition, it has been found that giving stress-induced inulin to mice can significantly increase the content of SCFAs in the cecum, particularly acetate and propionate [159], while reducing pro-inflammatory cytokines and corticosterone levels associated with stress [165]. Notably, the prebiotic bimuno-galacto-oligosaccharide (B-GOS) mitigates cognitive dysfunction in rats and markedly suppresses microglial activation, along with the expression of iNOS, CD68, CD32, SOCS3, and IL-6 [166]. Additionally, B-GOS has been shown to enhance β-diversity of the GM, promoting *Bifidobacterium* proliferation and the growth of anti-inflammatory microbiota [151]. Collectively, these findings suggest that prebiotics may exert beneficial effects on brain function via the MGBA, though the precise mechanisms remain incompletely elucidated.

#### 3.1.3. Synbiotics

According to the latest 2019 definition by the International Scientific Association for Probiotics and Prebiotics (ISAPP), synbiotics are defined as a combination of live microorganisms and substrates that may confer health benefits on the host [167]. The ISAPP indicates that synbiotics can be further divided into two subcategories. Complementary synbiotics include probiotics and prebiotics for the microorganisms produced by the human body itself, while synergistic synbiotics contain substrates that can be utilized by the co-administered microorganisms [8]. Synbiotics, a synergistic complex of probiotics and prebiotics, effectively compensate for the limitations of sole probiotic supplementation by significantly enhancing probiotic survival in the gastrointestinal environment. This formulation regulates the GM composition of the host, thereby potentially exerting beneficial effects on the CNS [168].

For example, a study demonstrated that a synbiotic combining poly-mannuronic acid with *Lactobacillus rhamnosus* GG exhibited significant neuroprotective effects in individuals with PD, highlighting its potential as an ideal oral therapeutic agent for PD [143]. Another randomized controlled trial revealed that fermented milk containing a multi-strain probiotic blend and prebiotic fibers outperformed placebo in alleviating constipation symptoms in PD patients [169]. Notably, a 12-week synbiotic intervention (Enterolactis Duo, containing *Lactobacillus paracasei* DG and the prebiotic fiber inulin) significantly improved gastrointestinal symptoms in constipated PD patients, suggesting potential efficacy of synbiotics in managing non-motor symptoms of PD [170].

In addition, a recent study has shown that a nicotinamide mononucleotide (NMN) synbiotic (a combination of NMN, Lactiplantibacillus plantarum CGMCC 1.16089, and lactulose) can significantly alleviate AD symptoms. The NMN synbiotic intervention has multiple positive effects on AD. Firstly, it significantly reduces the deposition of Aβ in the cerebral cortex and hippocampus. Secondly, the expression of tight junction proteins claudin 1 and ZO-1 is significantly upregulated, enhancing the integrity of the intestinal barrier. Finally, the NMN synbiotic reduces the expression of pro-inflammatory cytokines interleukin 1β (IL-1β), interleukin 6 (IL-6), and tumor necrosis factor alpha (TNF-α), and lowers the reactive oxygen species (ROS) level, indicating a reduction in oxidative stress. The reduction in Aβ deposition, enhancement of intestinal barrier function, decrease in neuroinflammation, and alleviation of oxidative stress suggest that the NMN synbiotic provides a promising therapeutic intervention for AD by regulating multiple pathological pathways [171].

Overall, synbiotics exert beneficial effects on the host by directly enhancing the abundance of probiotic bacteria as well as selectively stimulating the growth and metabolic activity of beneficial bacteria. These actions enhance intestinal barrier function and reduce neuroinflammation and oxidative stress, thereby promoting CNS protection [172]. For example, a novel synbiotic (Triphala and *L. plantarum*, *L. fermentum*, and *B. longum* subsp. infantis) has been found to reduce markers of inflammation, immunity, and oxidative stress by regulating the peroxisome proliferator-activated receptor gamma (PPAR-γ) pathway. At the same time, it reduces β-amyloid deposition and acetylcholinesterase activity in an AD transgenic human Drosophila model and enhances exercise ability and survival rate [173]. However, the specific metabolic pathways in the above studies have not been fully elucidated, and the intermolecular interaction mechanism has not been studied. In the future, experimental methods such as molecular docking simulation technology can be considered to further clarify the molecular mechanism of related signal transduction. Molecular docking simulation technology is a highly popular and well-established computational method. It has been widely used to understand the molecular interactions between natural organic molecules (preferably acting as receptors), such as enzymes, proteins, DNA, and RNA, and natural or synthetic organic/inorganic molecules (regarded as ligands), as well as to explore the mechanisms of biomolecules [174].

#### 3.1.4. Postbiotics

Postbiotics are a relatively new concept that was proposed and defined by the ISAPP in 2021 as “preparations of inanimate microorganisms and/or their components that confer health benefits on the host” [175]. In other words, postbiotics include not only intact inactivated microorganisms (excluding live microorganisms) but also structural fragments of microorganisms. With the deepening of research, the metabolic and secreted products generated by microorganisms during growth and fermentation, such as SCFAs, secondary bile acids, exopolysaccharides (EPS), and other substances, can also be classified as postbiotics [176,177].

Postbiotics have a positive effect on neurological diseases. They can prevent the damage and loss of dopaminergic neurons by increasing the level of dopamine (DA) and reducing the content of α-synuclein in the substantia nigra region. They also inhibit the aggregation of neurofibrillary tangles (NFTs) and reduce the deposition of amyloid-β peptide in the body, thus improving motor function defects [178].

In addition, postbiotics can actively promote the secretion of neurotrophic factors, further strengthening their protective effect on the nervous system [179]. For example, after giving specific postbiotic intervention to PD model mice, it was found that the dopamine level in the mouse brain was significantly increased compared with a control group, and symptoms such as bradykinesia and tremors were improved [178]. It was observed that children with ASD who consumed foods rich in postbiotics exhibited an increase in neurotrophic factor levels within their bodies. Additionally, symptoms such as social impairments and stereotyped behaviors showed varying degrees of improvement [180].

Moreover, postbiotics can also increase the production of inhibitory neurotransmitters such as GABA, helping to reduce anxiety and relieve related diseases such as anxiety disorders to a certain extent [181]. In chronic inflammatory diseases of the nervous system, such as multiple sclerosis, GABA can reduce the inflammatory response and protect nerves [182].

It is worth noting that numerous studies have shown that postbiotics also have excellent antioxidant activity, which can effectively scavenge excess free radicals in the body and reduce the damage caused by oxidative stress to the nervous system [179].

### 3.2. Mechanisms by Which Microecologics Exert Effects on Central Nervous System Diseases

**Figure 2:**
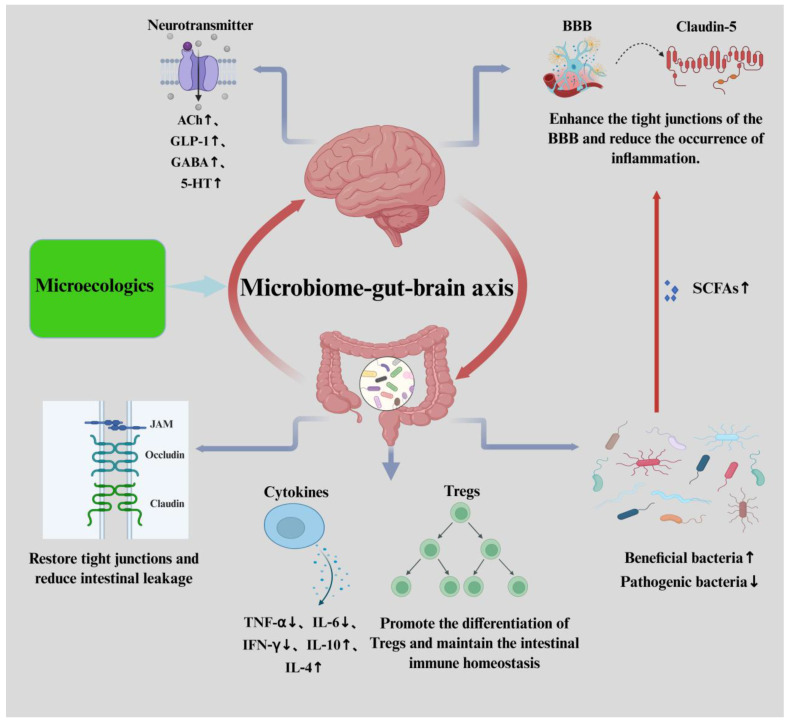
The regulatory mechanism of microecologics on central nervous system (CNS) diseases. Microecologics can improve CNS diseases by regulating the intestinal flora and its metabolites, intestinal barrier function, immune function, blood–brain barrier (BBB), and neurotransmitters. For instance, microecologics can increase the abundance of beneficial intestinal bacteria (such as *Lactobacillus* and *Bifidobacterium*), reduce the number of pathogenic bacteria, restore microbial diversity, and inhibit the colonization of pathogenic bacteria. Microecologics can also upregulate the expression of tight junction proteins (such as ZO-1 and occludin) in IEC, strengthen intestinal barrier function, and prevent pathogen invasion. At the same time, microecologics can upregulate the expression of tight junction proteins (such as occludin and claudin-5) in BBB endothelial cells by activating GPR41/43 receptors or inhibiting HDACs, thereby reducing BBB leakage. Microecologics promote the generation of Tregs, inhibit the release of pro-inflammatory factors such as tumor necrosis factor alpha (TNF-α), interleukin 6 (IL-6), and interferon gamma (IFN-γ), and instead promote the secretion of anti-inflammatory factors such as IL-10 and IL-4, balancing intestinal immune responses. In addition, there is evidence that microecologics can increase the concentration of neurotransmitters such as 5-hydroxytryptamine (5-HT), glucagon-like peptide 1 (GLP-1), and gamma-aminobutyric acid (GABA) in the brain, and can also activate the vagus nerve, release acetylcholine (ACh), and inhibit the activation of microglia, thereby improving the symptoms of CNS diseases. ↑: The content in the body increases; ↓: Decrease in body content.

As shown in Figure 2, microecologics improve central nervous system diseases through a variety of mechanisms, including enhancing intestinal barrier function, regulating the blood–brain barrier, regulating immune function, regulating neurotransmitter systems and the vagus nerve, and modulating the gut microbiome.

#### 3.2.1. Enhancement of Intestinal Barrier Function

The intestinal barrier is composed of the mucus layer, the epithelial barrier, and the vascular barrier of the intestine, which together provide excellent protection for the host. It can prevent harmful substances such as bacteria, viruses, toxins in the intestine from entering the human body and maintain the microecological balance and normal physiological functions in the intestine [183,184]. However, an imbalance in the GM may lead to intestinal barrier dysfunction (increased intestinal permeability, also known as the “leaky gut” phenomenon) [185], causing pathogenic microorganisms and their metabolic products to migrate to the portal vein and peripheral circulation system. This trans-barrier microbial translocation can activate a systemic immune response and ultimately induce central nervous system diseases such as ASD through neuroinflammatory pathways [185,186].

Tight junctions formed by tight junction proteins are crucial for maintaining the function of the intestinal barrier. They can prevent harmful substances in the intestine from entering the bloodstream, thereby reducing damage to the CNS [187]. Some studies have found that microecologics can interact with intestinal epithelial cells to promote the expression and assembly of tight junction proteins, thereby enhancing intestinal barrier function [188]. Research has shown that microecologics can exert beneficial effects on neurological diseases by enhancing intestinal barrier function. For example, some studies have shown that *Lactobacillus bifidus* can upregulate the expression of tight junction proteins (such as ZO-1 and occludin) in intestinal epithelial cells (IECs), strengthen intestinal barrier function, and effectively block the invasion of pathogens. In addition, microecologics can improve intestinal permeability, reduce the entry of endotoxins from the intestine into the blood, and decrease systemic inflammatory responses, thereby avoiding indirect damage to the nervous system by inflammatory factors. For example, studies have found that a symbiotic bacterium, *Bacteroides fragilis*, is considered to have the function of a probiotic. It can repair intestinal permeability and thereby alleviate ASD symptoms [189].

SCFAs are produced during the fermentation of dietary fiber by GM. These products play a vital role in restoring the integrity of the intestinal barrier, which is essential for reinforcing the mucus layer. When the human body ingests a relatively large amount of dietary fiber, the number of microorganisms in the intestine that can metabolize dietary fiber and produce SCFAs will increase accordingly. This change in the microbial community will in turn stimulate mucus secretion, thereby maintaining the good structure and integrity of the mucus layer [190,191]. Microecologics can also promote the production of SCFAs, indirectly enhancing the intestinal barrier function [192]. For example, a proof-of-concept trial conducted in patients with PD showed that prebiotic fiber intervention that promoted SCFA production reduced plasma zonulin, indicating an improvement in intestinal barrier integrity [193].

However, at present, the composition of GM varies greatly among different individuals (affected by factors such as genetics, diet, and environment). As a result, the specific targets of microecologics in enhancing intestinal barrier function have not been fully elucidated.

#### 3.2.2. Regulation of the Blood–Brain Barrier

The BBB is a multicellular vascular structure that separates the CNS from the peripheral blood circulation [194]. An intact BBB is crucial for constructing and maintaining a microenvironment that allows neuronal circuits to function properly. It can prevent harmful substances in the blood, such as bacteria, viruses, toxins, and some large molecules, from entering the brain tissue. It provides a stable and relatively independent internal environment for the brain, protecting the brain from external harmful substances and maintaining the normal physiological functions and neural activities of the brain [194]. However, when the normal composition of the GM is disturbed, it might significantly increase the permeability of the intestinal barrier and simultaneously enhance the immune activation response. This change leads to the occurrence of systemic inflammatory responses, which in turn may damage the BBB, promote the occurrence of neuroinflammation, and lead to neural damage [195].

Microecologics can regulate the expression and function of intercellular junction proteins, enhance the tight junctions of the BBB, and reduce its permeability, thereby protecting the BBB. For example, SCFAs such as butyrate can upregulate the expression of tight junction proteins such as occludin and claudin 5 in BBB endothelial cells by activating G protein-coupled receptors (such as GPR41/43) or inhibiting HDACs, reducing BBB leakage [196]. There is also evidence that some microecologics can protect the intestinal barrier by reducing the rate of epithelial cell apoptosis, improving intestinal function, and indirectly having a positive impact on the BBB [197].

On the other hand, intestinal inflammation can lead to the release of various inflammatory factors. These inflammatory factors can pass through the BBB via the bloodstream and reach the brain, triggering neuroinflammation. Microecologics can reduce the production and release of inflammatory factors by regulating intestinal immunity, decrease the levels of inflammatory factors in the bloodstream, and thus alleviate the damage of inflammatory factors to the BBB [198,199]. It is worth mentioning that the beneficial bacteria in microecologics can also inhibit the growth of harmful bacteria by competing for adhesion sites and producing antibacterial substances, reducing the occurrence of intestinal inflammation. For example, in patients with inflammatory bowel disease, intestinal barrier function is impaired, and the permeability of the BBB also increases. After supplementation with probiotics, intestinal inflammation is alleviated, intestinal barrier function is improved, and the permeability of the BBB is also reduced accordingly [200].

In addition, a substantial body of evidence suggests that metabolites derived from the GM play a crucial regulatory role in maintaining the integrity of the BBB [103,201,202]. For instance, TMAO is a GM-derived metabolite produced through the interaction of GM and the liver enzyme flavin-containing monooxygenase 3 (FMO3) [203]. TMAO can upregulate the expression of MMP-2 and MMP-9, enzymes that degrade key tight junction proteins such as occludin and ZO-1, leading to BBB leakage. Moreover, TMAO can activate the NLRP3 inflammasome, promoting the secretion of pro-inflammatory cytokines like IL-1β and IL-18 by BBB endothelial cells. Simultaneously, it upregulates vascular endothelial adhesion molecules (such as VCAM-1 and ICAM-1), facilitating the infiltration of peripheral immune cells into the CNS and exacerbating the progression of CNS diseases [203]. On the contrary, some metabolites produced by microecologics, such as SCFAs, possess anti-inflammatory properties. They can directly act on BBB endothelial cells, regulate intracellular signaling pathways, enhance the tight junctions of the BBB, and maintain its integrity [54]. For example, the beneficial effects of SCFAs on BBB epithelial cells have been observed in a mouse CNS model [196]. Although it has been demonstrated that the GM affects BBB permeability through metabolites or immune regulation, the specific regulatory details of the signaling pathways remain unclear. Future research is needed to elucidate the molecular mechanisms by which microbiota metabolites (such as SCFAs, TMAO, and tryptophan derivatives) regulate BBB tight junction proteins (such as claudin 5 and ZO-1).

#### 3.2.3. Regulation of Intestinal Immune Function

The GBA theory reveals the dynamic bidirectional interaction between the GM and the brain. Disruptions in its signaling pathways can directly lead to CNS dysfunction. Research has shown that the gut ecosystem is not only involved in digestion and absorption but also serves as the body’s largest immune organ, playing a significant role in brain development [105,204,205]. When the GM becomes dysregulated, it can disrupt the host’s immune-metabolic homeostasis and even influence the progression of CNS diseases. In the CNS, in addition to glial cells, resident immune cells (such as macrophages, CD8^+^ T cells, regulatory T cells, and other CD4^+^ helper T cell (Th) subsets) actively participate in innate and/or adaptive immune responses [206,207,208]. However, during the process of gut immune regulation, the effects of microecologics on the gut and the immune system have been widely recognized. They play multiple roles in gut responses and the immune system. For example, literature has demonstrated that *Lactobacilli* can regulate the direction of gut immune cells, promoting the generation of a large number of regulatory Tregs, inhibiting the release of pro-inflammatory factors such as TNF-α and IL-6, and instead promoting the secretion of anti-inflammatory factors such as interleukin-10 (IL-10), thereby balancing the gut immune response. Moreover, it guides the gut mucosal immune system to secrete immunoglobulin A (IgA) appropriately, which binds tightly to pathogens, ensuring that gut–brain axis signal transduction is not disrupted by excessive immune activity [209].

It is worth noting that the balance between Th1 and Th2 lymphocytes is crucial for immune regulation. Microecologics can induce the differentiation of regulatory T lymphocytes and the synthesis of anti-inflammatory cytokines, restoring the imbalance between Th1 and Th2 lymphocytes in the immune system [210]. These pieces of evidence seem to indicate that microecologics are beneficial for maintaining the balance of gut immunity and preventing excessive or insufficient immune responses.

Recent studies have found that microecologics can stimulate the gut immune system by activating the aryl hydrocarbon receptor (AhR) pathway [211,212]. Activation of the AhR pathway can affect the differentiation and function of various immune cells. The AhR signal can regulate the balance of T-cell subsets, promote the differentiation of regulatory Tregs, inhibit the overactivation of pro-inflammatory T cells, and maintain gut immune homeostasis. AhR can also affect the antibody secretion of B cells, promote the production of IgA, and enhance the mucosal immune defense of the gut [213].

A study showed that microecologics can enhance the expression of anti-inflammatory cytokines while reducing the expression of pro-inflammatory cytokines [214]. For example, in a mouse model immunized with multiple sclerosis peptides, the probiotic *Streptococcus thermophilus* ST285 significantly increased the expression of anti-inflammatory cytokines IL-4, IL-5, and IL-10 and decreased the levels of pro-inflammatory IL-1β and IFN-γ [215].

In addition, microecologics can also regulate the functions of various immune cells, including T cells, B cells, macrophages, dendritic cells, and innate lymphoid cells [216,217]. For instance, SCFAs have been proven to significantly promote the differentiation process of T cells and provide necessary support for the functional expression of regulatory B cells, thereby effectively limiting the occurrence and development of inflammation [218,219,220]. SCFAs can stimulate the production of retinoic acid in the gut, thereby inhibiting the differentiation of T helper 17 (Th17) cells and promoting the proliferation of Treg cells, and have a beneficial effect on neuroinflammation [221] and in preclinical models of MS [222]. However, the pathways through which microecologics regulate the differentiation and function of immune cells remain unclear. Further research is needed.

#### 3.2.4. Regulation of Neurotransmitters and the Vagus Nerve

Neurotransmitters are chemical substances that transmit information between neurons or between neurons and effector cells. Neurotransmitters mainly include 5-HT, GABA, acetylcholine (ACh), GLP-1, etc. [223]. They play a crucial role in the process of neural signal transmission. They can stimulate the vagus nerve endings on the intestinal wall, thus influencing brain functions such as mood regulation and cognitive function [223]. The vagus nerve is a component of the parasympathetic nervous system and a crucial pathway for neural communication between the CNS and the GM [125,224].

The intestine is the main site of 5-HT synthesis (about 95% of 5-HT in the human body is synthesized here). Microecologics can optimize the intestinal environment, create suitable conditions for intestinal enterochromaffin cells, affect the activity of related enzymes, and regulate the synthesis and release of 5-HT. Subsequently, 5-HT transmits signals to the brain through the blood circulation or the vagus nerve, participating in mood and cognitive regulation. A study has shown that the co-administration of the FOS and GOS increased the concentration of 5-HT in the brains of APP/PS1 mice. In addition, the administration of the prebiotic polymannuronic acid led to an increase in 5-HT and its metabolite 5HIAA in the striatum of MPTP-induced mice. This also indicates that prebiotics seem to be able to regulate the synthesis and release of 5-HT, and thus affect the brain through the vagus nerve [225].

GABA is the main inhibitory neurotransmitter in the central nervous system and can bind to the GABA receptors on the vagus nerve endings. When the binding occurs, it changes the excitability of the vagus nerve [226]. Some microecologics can also regulate the metabolism of GABA in the intestine, change the abundance of GABA-metabolizing bacteria, or induce intestinal cells to produce more GABA, optimizing the level of GABA in the intestine. After GABA enters the brain, it regulates the excitability of neurons and alleviates emotional problems such as anxiety and depression. For example, a recent randomized controlled trial found that compared with PD patients who received only the conventional regimen, the co-administration of probiotics and the conventional regimen in PD patients increased the abundance of species-level genomic bins (SGBs) involved in GABA synthesis and decreased the abundance of SGB encoding GABA degradation [227]. This precise regulation of the GM may have a positive impact on the neurological function, motor symptoms, and disease progression of patients with PD by affecting the metabolic balance of GABA.

In addition, ACh plays a crucial role in both the peripheral and central nervous systems. The activation of the “cholinergic anti-inflammatory pathway” mediated by the binding of ACh to α7 nicotinic acetylcholine receptors (nAChRs, the most abundant nAChR subunit in mitochondria) [228] is one of the potential mechanisms for controlling inflammation [229]. Acetylcholinesterase (AChE), the primary enzyme responsible for hydrolyzing ACh, shows decreased activity as the symptoms of AD advance [230]. The roles of AChE in regulating the activities of other proteins, regional cerebral blood flow, tau phosphorylation, and the amyloid cascade reaction may influence the progression rate of AD [230]. Microecologics can regulate the gene expression of AChE, reduce the decomposition of ACh, and improve the neuroinflammatory state of the brain. For example, the probiotic Lactobacillus plantarum restored the level of ACh in the brains of D-galactose-induced AD rats by reducing the activity of AChE. Similarly, another study found that the prebiotic FOS from Morinda officinalis prebiotics could also restore the ACh level in the brains of galactose-induced AD rats by reducing the AChE level [141,231].

It is worth mentioning that GLP-1 is an incretin hormone produced by enteroendocrine cells (EECs) after the ingestion of nutrients. Previous evidence has shown that probiotics and prebiotics can stimulate the secretion of GLP-1 and upregulate the expression of GLP-1 receptors in the brain, thereby exerting neuroprotective effects on the CNS [232,233]. The above evidence indicates that microecologics can regulate neurotransmitters and the vagus nerve through the MGBA, thereby alleviating CNS diseases. However, there is a lack of direct evidence on how microecologics affect CNS function through the vagus nerve, and further studies are needed.

#### 3.2.5. Regulation of Gut Microbiota

A substantial body of studies have identified a strong association between GM dysbiosis and various CNS disorders [180,234,235]. For example, the GM of patients with AD exhibits significant alterations compared with healthy controls, including increased *Erysipelotrichia*/*Erysipelotrichaceae* and decreased *Eubacterium rectale* and *Bacteroides fragilis Erysipelotrichia*/*Erysipelotrichaceae*, can promote the occurrence of inflammation, which may be related to the peripheral inflammatory state of AD [236]. In children with ASD, *Bifidobacterium*, *Akkermansia muciniphila*, and *Alistipes* levels are lower than in healthy controls, whereas *Lactobacillus*, *Bacteroides*, *Prevotella*, and *Parabacteroides* are elevated [237,238,239]. These changes in the composition of GM lead to decreased diet quality and nutrient deficiency in children with ASD [240]. In addition, a small longitudinal study found that MS patients harbor elevated *Pseudomonas*, *Haemophilus*, *Blautia*, and *Dorea* compared to healthy individuals, with reduced *Parabacteroides*, *Adlercreutzia*, and *Prevotella* [30]. This may exacerbate systemic inflammation in MS. Some studies reported that microecologics can intervene in the pathological process of CNS diseases by regulating the GM. This includes restoring microbial diversity, inhibiting the colonization of pathogenic bacteria, and promoting the functional activity of beneficial bacteria [241,242,243].

Firstly, microecologics can directly increase the proportion of beneficial bacteria in the gut, thereby improving the microbiota structure and subsequently alleviating CNS diseases. For example, fucoidan gum, which is a polysaccharide, also known as sulfated fucoidan gum (a common prebiotic that combines uniquely with sulfate groups), can significantly increase the abundance of beneficial bacteria, including *Ruminococcaceae* and *Lactobacillus*, and reduce pathogenic bacteria (especially *Peptococcus*) in the colon of diabetic mice (DM) [244]. Studies have shown that during the treatment of nerve damage, MOS can increase the number of *Lactobacillus* while reducing the number of *Helicobacter pylori*. Furthermore, MOS induced an increase in butyrate production, which significantly reduces the inflammatory response and strengthening the function of the immune system [10,245].

Although rapid and effective regulation of the structure and function of the microbiota is crucial for providing timely health benefits, the long-term colonization of the microbiota is also a key issue that must be considered when applying microecologics. There are various microbial adhesion sites on the surface of intestinal epithelial cells. Microecologics can prevent harmful bacteria from colonizing the intestinal epithelium by competing with harmful bacteria for adhesion sites. For example, one study showed that when *Bacillus subtilis* (a probiotic strain) was introduced into the gut of a rat model of PD, it increased the concentration of beneficial bacteria (namely *Bifidobacterium*) and decreased the concentration of *Escherichia coli* in the rats [246]. This effect was attributed to intense competition for nutrients and adhesion sites.

Studies have also reported that microecologics can alleviate CNS diseases by regulating the GM and ameliorating gastrointestinal defects. For example, a study has shown that oral administration of *Bacteroides fragilis* (a symbiotic bacterium that can be regarded as a probiotic) can correct the intestinal epithelial permeability caused by dysbiosis, thereby regulating the levels of various metabolites, especially 4-EPS, and improving the symptoms of ASD [247]. Although gut microbes are known to affect the CNS through immune, metabolic, and neural pathways, the specific mechanisms have not been elucidated, which is not conducive to the development and application of targeted microecologics. Moreover, the clinical effect of microecologics is unstable. Therefore, further research is needed to establish unified standards for microecologics in terms of types, doses, and courses of treatment. Although the potential of microecologics to improve CNS diseases by regulating the gut–brain axis has been confirmed, the limited duration and individual differences in their therapeutic effects restrict their intervention effects. Exercise, a non-drug intervention method, can enhance the function of the gut–brain axis and the bioavailability of bacterial metabolites, and may form a synergistic effect with microecologics.

## 4. The Influence of Exercise on Central Nervous System Diseases

### 4.1. Modulatory Effects of Exercise on Central Nervous System Diseases

A growing body of studies have shown that exercise is one of the most validated non-invasive strategies for addressing CNS disorders. Exercise can protect or improve CNS diseases (such as ASD, AD, PD, etc.) by regulating intestinal microbiota and its metabolites, reducing inflammatory response, and improving neuroplasticity [248,249,250].

First of all, exercise can have a positive impact on the microbiota. For example, studies have found that exercise can increase the abundance of butyrate-producing bacteria, such as *Roseburia hominis*, *Faecalibacterium prausnitzii*, and *Ruminococcaceae* [251,252,253,254,255,256]. Butyrate has the ability to inhibit histone deacetylases, and has an impact on gene regulation, immune regulation, cell differentiation, intestinal barrier regulation, oxidative stress reduction and intestinal peristalsis regulation, which in turn can have a positive effect on the CNS [257].

Secondly, exercise has been proven to induce anti-inflammatory effects [258], thus playing an active role in the prevention and treatment of CNS diseases. For example, an experimental study in a mouse model of obesity-associated neuroinflammation has shown that exercise protects against obesity-induced white matter damage by inhibiting neuroinflammation and vascular dysfunction [259]. Exercise significantly increases the mRNA expression of ATP-binding cassette transporter A1 (ABCA1). ABCA1 is a key protein involved in cholesterol reverse transport. It participates in transporting intracellular cholesterol to apolipoprotein A-I (apoA-I) to form high-density lipoprotein (HDL), promoting the clearance of Aβ in the brain [260].

Thirdly, exercise can also promote the secretion of neurotrophic factors in the body. These factors not only contribute to the growth, survival, and differentiation of neurons and enhance brain plasticity but also regulate intestinal function through vagus nerve feedback, forming a virtuous cycle and improving the social and cognitive deficits of patients with ASD. Moreover, the neural signaling triggered by exercise can regulate the function of the HPA axis, reducing the excessive secretion of stress hormones such as cortisol and alleviating the damage caused by long-term stress to the CNS [261,262]. A pilot randomized controlled trial demonstrated that exercise could alleviate the overall decline in cognitive function among elderly individuals with mild-to-moderate AD [263]. Moreover, a systematic review and meta-analysis indicated that endurance or aerobic exercise improved the functional scores of patients with ALS [264]. It is worth noting that a study has shown that for patients with PD, undergoing 8-week high-intensity aerobic exercise in either a mandatory or voluntary mode improved their PD symptoms and hand dexterity and enhanced the function of the CNS [265]. These pieces of evidence suggest that exercise has a positive impact on CNS diseases. Apart from exercise, the Mediterranean diet (MD) is the best-studied and most evidence-based diet for the prevention of cardiovascular disease and several other chronic diseases, including neurodegenerative diseases [266,267,268]. However, since it is outside the scope of this article, only the concept is mentioned without relevant overview. If interested, researchers can learn more about this intervention.

### 4.2. Possible Mechanisms by Which Exercise Exerts Its Effects on Central Nervous System Diseases

**Figure 3:**
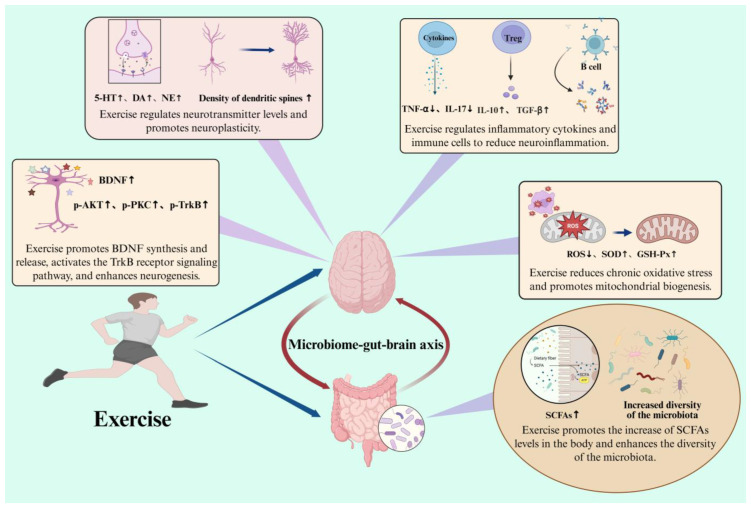
The mechanism of exercise in regulating central nervous system (CNS) diseases. Exercise improves CNS diseases by regulating the immune system, inhibiting neuroinflammation, reducing oxidative stress, and modulating neurotransmitters, neurotrophic factors, and the gut microbiota. For instance, exercise suppresses neuroinflammation by lowering the levels of pro-inflammatory cytokines (such as tumor necrosis factor alpha (TNF-α), interleukin 1β (IL-1β)) and upregulating the expression of anti-inflammatory cytokines (such as transforming growth factor β (TGF-β), interleukin 10 (IL-10)) and by modulating the activity of T cells and B cells to improve CNS diseases. Additionally, exercise exerts neuroprotective effects in CNS diseases by reducing chronic oxidative stress levels and through stimulating mitochondrial biogenesis and upregulating the autophagy pathway. Moreover, exercise regulates neurotransmitters and neurotrophic factors, increasing the levels of 5-hydroxytryptamine (5-HT), dopamine (DA), norepinephrine (NE), and brain-derived neurotrophic factor (BDNF) in the body, promoting elevation in phosphorylated protein kinase B (p-AKT), phosphorylated protein kinase C (p-PKC), and phosphorylated tyrosine kinase receptor B (p-TrkB) levels, thereby inhibiting neuroinflammation, enhancing synaptic plasticity, and regulating mood. Finally, exercise increases the diversity of the microbiota and the balance between beneficial and pathogenic bacteria and raises the levels of short-chain fatty acids (SCFAs) in the body, exerting a positive impact on CNS diseases. ↑: The content in the body increases; ↓: Decrease in body content.

As illustrated in Figure 3, exercise exhibits beneficial effects on central nervous system diseases through a variety of mechanisms. These include counteracting neuroinflammation and regulating immune responses, exhibiting antioxidant properties, modulating neurotransmitter levels, influencing neurotrophic factors, and regulating the composition of intestinal microbiota.

#### 4.2.1. The Anti-Neuroinflammatory and Immunomodulatory Effects of Exercise

Many neurological diseases, such as AD, are accompanied by an inflammatory response. As a common neurodegenerative disease, in the brains of AD patients, a large amount of β-amyloid protein accumulates, triggering excessive activation of microglia, which in turn generates a series of inflammatory factors, such as IL-1β, TNF-α, etc. These inflammatory factors not only damage the synaptic connections between nerve cells, impeding the normal transmission of nerve signals, but also induce apoptosis of nerve cells, accelerating the decline in cognitive function [42].

Exercise, an effective intervention measure, may improve CNS diseases by inhibiting neuroinflammation. For example, in an experimental EAE model, preventive swimming training reduced the levels of pro-inflammatory cytokines (such as IFN-γ and IL-17) and simultaneously increased the levels of anti-inflammatory cytokines (such as TGF-β and IL-10), significantly improving symptoms in MS mice [269,270]. Studies have found that exercise seems to stimulate the secretion of growth hormone and prolactin [271]. Growth hormone can reduce the levels of pro-inflammatory cytokines (such as TNF-α and IL-1β) and upregulate the expression of anti-inflammatory cytokines (such as IL-10), thereby inhibiting neuroinflammation [272]. Prolactin can inhibit the excessive activation of macrophages and microglia, reducing their release of pro-inflammatory cytokines. For instance, it can inhibit the release of TNF-α and IL-1β by microglia, thus alleviating the damage of neuroinflammation to neural tissue [273]. It is certain that exercise is able to ameliorate CNS disease by inhibiting or reducing neuroinflammation. However, so far, the relevant mechanism is still unclear, and more studies are needed to verify the relevant mechanism.

A recent study used 2D gel electrophoresis combined with liquid chromatography–tandem mass spectrometry for protein identification. In a human study, blood tests were conducted on subjects before high-intensity interval exercise and at 5 min and 1 h after the exercise. The tests revealed significant changes in the abundance of 20 isolated serum proteins [274]. Some proteins in the serum showed a significant increase, including alpha-1 antitrypsin, fetuin A, vitamin D-binding protein, histidine-rich glycoprotein, apolipoprotein J (clusterin), apolipoproteins E and A1, and immunoglobulin J chain. Other proteins decreased, including immunoglobulin heavy constant α1, immunoglobulin k constant, and β-2-glycoprotein 1 [274]. Among the increased proteins, serine protease inhibitors, apolipoproteins, and immune system proteins have a broad anti-inflammatory effect and are involved in cardioprotection, neuroprotection, and lipid clearance [274]. This research provides further evidence that exercise protects neural tissue by inhibiting or alleviating neuroinflammation, closely associating with the improvement of CNS diseases through exercise. However, further research is needed to elucidate the mechanisms by which exercise provides neuroprotection for CNS diseases through specific signaling pathways.

In addition, exercise can improve CNS diseases by regulating the immune system. Specifically, moderate exercise can enhance the activity of T cells, promoting their ability to recognize and eliminate pathogens. It can also regulate the function of B cells, enhancing antibody production and thus improving the body’s resistance to infection [275,276]. The regulatory effect of exercise on the immune system has also been verified in different animal models. For example, in the EAE model, exercise has been proven to alleviate the symptoms and pathological changes of MS by regulating the activity of T cells and B cells. Moreover, in other demyelinating models, such as cuprizone (CPZ) and lysophosphatidylcholine (LPC) models, exercise has also been found to regulate the activity of microglia, reducing inflammation and demyelination damage [277].

#### 4.2.2. The Antioxidant Effect of Exercise

Previous studies have shown that oxidative stress is an important factor leading to neuronal cell death in CNS diseases such as AD and PD [278]. Oxidative stress results from the excessive generation of free radicals (such as reactive oxygen species, ROS) and an imbalance in the antioxidant system, leading to damage to lipids, proteins, and DNA [279]. Mitochondria are the main sites of energy production within cells and also important sites for the generation of free radicals. Through exercise, chronic oxidative stress can be reduced and mitochondrial biogenesis can be promoted, thereby reducing the generation of free radicals. These adaptive changes not only delay aging but also have a protective effect on ROS-related diseases such as CNS diseases [279,280]. For example, a study has shown that exercise can optimize respiratory efficiency and increase the activity of electron transport chain (ETC) complexes (I, III, IV), thus reducing ROS generation [281].

It is worth noting that exercise can effectively reduce protein and lipid damage caused by oxidative stress. For example, a study found that exercise training significantly reduced the levels of protein carbonylation and lipid peroxidation in the spinal cord of EAE mice [282]. Studies have shown that exercise can significantly reduce the level of chronic oxidative stress in patients with PD and enhance cellular repair capacity by stimulating mitochondrial biogenesis, upregulating autophagy pathways, and other means [283,284]. In addition, exercise can also significantly improve the antioxidant capacity of EAE mice by enhancing the activity of antioxidant enzymes. For instance, exercise training significantly increased the activity of glutathione peroxidase (GPx) and restored the content of glutathione (GSH). These antioxidant enzymes play a crucial role in scavenging free radicals and maintaining redox balance [282].

Previous studies have also reported the reduction of oxidative stress-related parameters in humans [284] and animal models [285] that underwent aerobic and resistance training, respectively. The study found that compared with the control group, the level of hydrogen peroxide (an oxidizing compound) in the blood of PD patients who underwent 8 weeks of resistance training was significantly reduced [284]. In a study on Wistar rats induced with PD, aerobic training (for 8 weeks) promoted an increase in the levels of antioxidant enzymes (superoxide dismutase and catalase) and reduced oxidative damage to lipids and proteins [285]. Notably, the appropriate intensity and volume of exercise are crucial factors influencing the antioxidant effect. Some studies have indicated that high-intensity and/or prolonged-duration exercise may also induce oxidative stress in the human body. Further research is necessary to clarify this controversial issue.

#### 4.2.3. Exercise Regulates Neurotransmitters and Neural Plasticity

Recent studies have revealed that as the basic units of brain information transmission, the synthesis and release regulation of neurotransmitters are not only directly related to the mood improvement effect mediated by exercise but also achieve the optimal reorganization of neural circuits by regulating synaptic plasticity, promoting the differentiation of neural stem cells, and other pathways. 5-HT, DA, and norepinephrine (NE) are the three main monoamine neurotransmitters regulated by exercise. Exercise can increase their levels in the body [286,287].

As a neurotransmitter, 5-HT has a significant impact on emotional states, emotion-related behaviors, and the sleep–wake cycle [288]. Branched-chain amino acids (BCAAs) share the L-type amino acid transporter 1 (LAT1) with tryptophan, and there is a competitive uptake relationship between them [289]. Exercise promotes an increased uptake of BCAAs by muscles. This change increases the probability of tryptophan crossing the blood–brain barrier and entering the brain, thereby increasing the level of 5-HT in the brain [290]. An elevated level of 5-HT can effectively relieve depression and anxiety, thus improving the CNS [290]. For example, a study has shown that compared with sedentary rats, the level of 5-HT in the hippocampus of rats subjected to 7 days of high-intensity treadmill exercise increased significantly [291]. However, the regulatory effects of different types and intensities of exercise on the 5-HT system vary, and the relevant mechanisms are not yet clear. Further exploration is needed in the future.

Secondly, DA is closely related to pleasure, motivation, and motor control and plays a crucial role in regulating immune functions related to T-cell activation and inflammation [292]. Pathologically low levels of DA may lead to various diseases, such as schizophrenia, ADHD, bipolar depression, addiction, and PD [293]. Studies have found that exercise can upregulate the effective concentration of DA in the brain [294]. Moreover, exercise can enhance the binding affinity between DA and its receptors, stimulate DA synthesis through the calcium/calmodulin-dependent system, strengthen related behaviors and memory, and thus alleviate CNS disease symptoms [294].

In addition, exercise has a significant impact on NE. An increasing body of evidence suggests that NE can eliminate oxidative stress, attenuate neuroinflammatory responses in neurons and glial cells, reduce the activity of neurons and glial cells, promote autophagy, and improve the apoptotic response to various injuries [295,296,297,298,299]. At the same time, it is also beneficial for the treatment of CNS diseases because it improves the production of neurotrophic factors, promotes neuronal survival, and plays an important role in the regulation of adult neurogenesis [300]. A report has stated that exercise can upregulate the level of NE in the brain and reduce depressive-like behaviors [301]. Exercise can promote the increase of NE level in the brain, thereby inhibiting neuroinflammation, enhancing synaptic plasticity, and regulating emotion to alleviate CNS diseases. However, the specific mechanism needs to be further verified.

#### 4.2.4. Exercise Regulates Neurotrophic Factors

Neurotrophic factors are growth factors belonging to the neurotrophic factor family. Among them, BDNF is a well-studied neurotrophic factor. BDNF plays a crucial role in neuronal plasticity, synaptic transmission, neuronal stress resistance, neuronal differentiation and maturation, and the activation of other supporting molecules such as NF-κB [302,303,304,305,306].

Studies have shown that in the early stages of AD, the levels of BDNF in the blood and brain of patients are relatively low, and the level of BDNF is positively correlated with cognitive function [307,308]. Research by Mattson et al. has demonstrated that exercise significantly increases the expression of BDNF in the brain [309]. In fact, a study found that in APP/PS1 mice, 12 weeks of treadmill running increased the level of BDNF in the hippocampus threefold while reducing Aβ deposition by 50% and significantly improving spatial memory [310]. A randomized controlled trial involving patients with mild cognitive impairment (MCI) showed that 6 months of aerobic exercise (three times a week, 40 min each time) increased the serum BDNF level by 24% and improved episodic memory and executive function [311]. These findings support the hypothesis that exercise-induced increases in BDNF production may contribute to delaying the progression of AD; however, further research is still needed.

In addition, studies have reported that in addition to reducing the levels of soluble Aβ in two regions, exercise can also increase the levels of brain-derived neurotrophic factor/tyrosine kinase receptor B (BDNF/TrkB) signaling molecules in the hippocampus and amygdala, including phosphorylated protein kinase B (p-AKT), phosphorylated protein kinase C (p-PKC), and phosphorylated tyrosine kinase receptor B (p-TrkB) [312]. This evidence also supports the notion that exercise can delay the progression of CNS diseases, especially AD, by enhancing the BDNF signaling pathway and promoting the clearance of Aβ.

It is worth mentioning that BDNF can also enhance synaptic connections between neurons, promote the formation of new synapses, and improve synaptic transmission efficiency. This enables the brain to transmit and process information more effectively during exercise, thereby improving the learning and memory of motor skills [313]. For example, in animal models of PD using 6-hydroxydopamine (6-OHDA) and MPTP, BDNF increased the survival rate of dopaminergic neurons, improving dopaminergic neurotransmission and motor performance [314,315,316]. In fact, exercise can increase the concentration of circulating BDNF, which may lead to an increase in neuroplasticity [317]. Animal experimental studies have shown that exercise can increase the expression levels of BDNF and tropomyosin receptor kinase B (TrkB). Exercise activates AMP-activated protein kinase (AMPK) and downstream signaling pathways, inducing the expression of peroxisome proliferator-activated receptor γ coactivator 1-α (PGC-1α). PGC-1α interacts with FNDC5 (fibronectin type III domain-containing protein 5), indirectly upregulating the expression of BDNF [318,319]. However, it is noteworthy that most current studies showing exercise can regulate neuroplasticity by upregulating the level of BDNF and may potentially improve CNS diseases have primarily been conducted using animal models. Thus, its effectiveness in clinical research remains unclear [320,321].

#### 4.2.5. Exercise Modulates Gut Microbiota

In recent years, the impact of exercise on the GM has emerged as a prominent topic of discussion in elucidating health benefits [254,257,322,323,324]. Generally, most studies reported a positive effect of exercise on the GM. This is mainly reflected in enhancing colonic health, increasing microbial diversity, and maintaining a balance between beneficial and pathogenic bacteria [325,326].

Exercise can promote microbial diversity and the enrichment of beneficial bacteria. As is well known, there are considerable differences in the composition and diversity of the microbiome between athletes and sedentary individuals. Studies have found that the alpha diversity of the GM in athletes is significantly higher than that in non-athletes, and the health-related microbiota (such as SCFAs-producing bacteria) is more abundant [327]. However, it remains unclear whether it is the better microbiota structure after exercise that makes athletes superior to non-athletes or whether a better microbiota structure shapes excellent athletic performance. More research is needed to further explore this issue. A study found that endurance (SE) training significantly increased the diversity and richness of the GM in the EAE mouse model and reduced the Firmicutes/Bacteroidetes (F/B) ratio. At the phylum level, the *Bacteroidetes* significantly increased while the *Firmicutes* decreased. At the genus level, SCFA-producing bacteria such as *Akkermansia* in the SE group were significantly enriched [328]. It is certain that the increase in the abundance of SCFA-producing bacteria leads to an increase in the level of SCFAs in the body. SCFAs can inhibit the excessive activation of microglia and reduce the release of pro-inflammatory factors (such as IL-1β and TNF-α) [99], thereby having a positive impact on CNS diseases.

Furthermore, exercise may also reduce the potential harm of pathogenic bacterial communities by increasing the frequency of intestinal peristalsis, reducing the retention time of contents and decreasing the contact between pathogens and the gastrointestinal mucus layer and the circulatory system. A recent study using a germ-free mouse model confirmed the causal role of exercise: after transplanting the microbiota of exercising mice into germ-free mice, their intestinal morphology, inflammatory status, and resistance to induced colitis were all superior to those of mice transplanted with the microbiota from the sedentary control group [251]. However, the relevant mechanisms are mainly based on animal models, and there are relatively few human clinical studies. There is also inconsistency in existing research, which not only increases the difficulty of systematic analysis but also limits the translation of findings into human research [324]. Moreover, some studies have shown that intense exercise can lead to increased intestinal permeability, gastrointestinal damage, and mild endotoxemia [329,330,331]. Therefore, the development of standardized experimental protocols (such as exercise intensity, duration, selection of gastrointestinal regions, dietary control, and diversity assessment indicators) has become an urgent issue to be addressed. Exercise can significantly improve the pathological process of CNS diseases by regulating immunity and oxidative stress. However, some preclinical studies have found that the combined intervention of exercise and microecologics for CNS diseases and other chronic diseases is more effective than either intervention alone. Therefore, exploring intervention methods where microecologics and exercise work synergistically has clinical translational potential and can provide innovative strategies for the treatment of CNS diseases.

## 5. The Possibility of Combined Intervention of Microecologics and Exercise on Central Nervous System Diseases

Microecologics have emerged as a promising strategy to improve CNS diseases by regulating the composition of the GM and its metabolites (such as SCFAs and tryptophan derivatives). Exercise intervention, a low-cost and non-invasive therapy, has been proven to delay the progression of CNS diseases through multiple pathways, such as promoting the secretion of neurotrophic factors, enhancing synaptic plasticity, and inhibiting chronic inflammation. However, although separate studies on microecologics and exercise have achieved encouraging results, the progress of research on combined interventions of the two remains relatively limited. Notably, both interventions can positively affect CNS diseases by regulating the composition of the GM and its metabolites through the MGBA. Therefore, it is highly anticipated that the combined intervention of the two can produce additive or synergistic effects through the MGBA, thereby more comprehensively and effectively improving CNS symptoms. In this section, we summarize the existing intervention studies on the combination of exercise and microecological regulators, not limited to CNS diseases, but aiming to explore the possibility of the combined intervention in CNS diseases.

The mechanisms underlying the combined application of microecologics and exercise in CNS diseases may involve multiple aspects, such as regulating the balance of the GM, influencing microbial metabolites, regulating neurotransmitter synthesis, and affecting neuroimmune responses. However, at present, relevant research progress is relatively limited, and we can only conduct a limited discussion based on existing research findings. For example, a study found that the combined effect of exercise and probiotics, two intervention measures, reduced the number and area of amyloid plaques in the hippocampus of mice. The combined action of the two can significantly delay the progression of AD in male APP/PS1 mice [15]. This study also found that the effects of exercise or probiotic treatment alone on improving spatial memory were limited. However, the combined effect of these treatments can indeed significantly improve brain performance. In this study, results of the Morris water maze test showed that during a 4-day training period, the latency of the combined-treatment group (APP/PS1-Ex-Pr) mice in locating a hidden platform shortened significantly day by day, indicating that their spatial learning and memory abilities were significantly better than those of the single-treatment groups (APP/PS1-Ex or APP/PS1-Pr). The results of the Y-maze spontaneous alternation test showed that compared with the control group, the number of alternations in the combined treatment group mice increased significantly (reflecting working memory ability), indicating the recovery of hippocampus-dependent cognitive functions. However, the improvement in the single-treatment groups was relatively small and did not reach a significant level. Similarly, the results of the open-field test showed that compared with the control group, the combined-treatment group was significantly better than the single-treatment groups [15]. These findings suggest that the microbiota regulation induced by the combined action of exercise and probiotics is important for alleviating the progression of AD.

In addition, some studies have found that the combined effect of exercise training and probiotic supplementation significantly increased the level of NRF-2 in the liver [332]. NRF-2 is one of the key participants in the cellular defense mechanism that activates antioxidant response element (ARE) genes (including SOD) to resist oxidative stress. As oxidative stress plays a crucial role in the occurrence and development of CNS diseases, it is reasonable to speculate that the combined effect of exercise and microecologics may improve the development of CNS diseases by reducing oxidative stress injury and inhibiting neuroinflammatory response. Although the results are encouraging, human studies are needed before definitive conclusions can be made about the effectiveness of combined interventions. To develop new therapeutic strategies, we must determine the translatability of these findings and generate high-quality data from preclinical and clinical studies to translate combined-intervention studies into clinical practice. In addition to the mechanisms already discovered, we can further explore the potential mechanisms of the combined application of microecologics and exercise in CNS diseases in terms of such parameters as neuroendocrine regulation, blood–brain barrier regulation, glial cell regulation, and mitochondrial function regulation. These new research directions are expected to provide more theoretical evidence for treatment and intervention in CNS diseases and promote the further development of related fields.

## 6. Conclusions

Targeted intervention strategies based on the MGBA offer new perspectives for the prevention and treatment of CNS diseases. Microecologics and exercise interventions, two non-pharmacological approaches, have a positive impact on the GM and brain function through the MGBA. The possibility of their synergistic effects has gradually become a research hotspot. However, current research still has several limitations. Firstly, more comprehensive studies that incorporate techniques such as metagenomics, metabolomics, proteomics, and single-cell transcriptomics are essential to elucidate the specific mechanisms by which the combined effects of microecologics and exercise influence CNS diseases through the MGBA. Secondly, large-sample, multi-center, long-term follow-up clinical studies are required to clarify the efficacy, safety, and optimal intervention regimens of these approaches in different types of CNS diseases. Most current research is based on animal models or small-sample clinical trials, and the small samples and short intervention periods limit the reliability and generalizability of the research results. Large-scale randomized controlled trials should be conducted to evaluate the synergistic effect of microecologics combined with exercise on CNS diseases, with a particular focus on long-term safety (such as the risk of bacterial resistance). Thirdly, standardized strain selection and dosage regimens of microecologics (such as the types of probiotics and prebiotics) are required. Additionally, a consensus must be reached regarding the intensity, frequency, and form (aerobic/resistance) of exercise interventions. Finally, personalized combined-intervention regimens should be developed based on patient characteristics (such as microbiota typing and metabolic phenotypes) and disease types (such as specific strains + customized exercise prescriptions).

In conclusion, the combined intervention of microecologics and exercise is a promising strategy for central nervous system diseases. However, further basic mechanism research is needed to deeply explore the specific molecular pathways and cellular mechanisms by which microecologics and exercise affect the nervous system, providing a more solid theoretical foundation for this intervention measure.

## Figures and Tables

**Table 1:** The characteristics of gut microbiota in neurological diseases and intervention strategies.

Types of Diseases	Main Symptoms	Model	Gut Microbiota Alterations	Intervention Strategies	References
AD	Cognitive impairment, neuropsychiatric symptoms, behavioral disorders	Adults	↑ Bacteroidetes, Bacteroidaceae, *Bacteroides*, Rikenellaceae, *Alistipes*, *Blautia*, *Bilophila* ↓ Actinobacteria, Firmicutes, Bifidobacteriaceae, *Bifidobacterium*, Ruminococcaceae, Turicibacteraceae, Clostridiaceae, *Dialister*, *Turicibacter*, *Adlercreutzia*	*Bifidobacterium* (Microecologics)	[20]
			↑ Proteobacteria, Gammaproteobacteria, Enterobacteriales, Enterobacteriaceae ↓ Firmicutes, Clostridiaceae, Lachnospiraceae, *Ruminococcaceae*, Ruminococcus, *Blautia*	Supplementing the flora that produces short-chain fatty acids	[21]
		Mice	↑ *Clostridium pasteurianum*, *Clostridium innocuum*, *Clostridium beijerinckii*, Firmicutes ↓ *Bacteroides ovatus*, *Bacteroides dorei*, *Bacteroides vulgatus*, Bacteroidetes	FMT	[22]
			↑ Bacteroidales ↓ *Akkermansia*, *Parabacteroides*	*Bacteroides fragilis* (Microecologics); FMT	[23]
PD	Cognitive impairment, gait disorder	Adults	↑ *Bifidobacterium dentium*, *Actinomyces oris*, *Streptococcus mutans*, *Lactobacillus fermentum*, *Escherichia coli*, *Klebsiella* spp., *Clostridium leptum*, *Enterococcus faecium* ↓ *Roseburia intestinalis*, *Blautia wexlerae*, *Faecalibacterium prausnitzii*, *Eubacterium rectale*, *Prevotella copri*, *Roseburia, Eubacterium*, *Ruminococcus*	/	[24]
			↑ *Ralstonia*, Oxalobacteraceae, *Akkermansia*, *Bacteroides*, *Oscillospira*, Proteobacteria, Verrucomicrobia ↓ *Faecalibacterium*, *Dorea*, Coprobacillaceae, *Blautia*, *Coprococcus*, *Roseburia*, Firmicutes	Supplementing butyrice-producing bacteria	[25]
		Mice	↑ Phylum Proteobacteria, Order Turicibacterales, Order Enterobacteriales ↓ Phylum Firmicutes, Order Clostridiales.	FMT	[26]
			↓ Verrucomicrobiae	FMT	[27]
ASD	Social communication deficits, restricted interests/repetitive behaviors	Children	↑ *Lactobacillaceae*, Bifidobacteraceae, Veillonellaceae, Erysipelotrichaceae, Enterococcaceae, Desulfovibrionaceae, *Lactobacillus*, *Bifidobacterium*, *Megasphaera*, *Mitsuokella*, *Klebsiella* ↓ Prevotellaceae, *Prevotella*, *Faecalibacterium*, *Roseburia*	*Lactobacillus* (Microecologics)	[28]
		Mice	↑ Lachnospiraceae, *Akkermansia*, *Sutterella* ↓ *Bacteroides*, *Parabacteroides*, Bacteroidetes	Taurine and 5AV	[29]
MS	Depression, fatigue, pain, cognitive impairment	Adults	↑ *Pseudomonas*, *Mycoplasma*, *Blautia*, *Dorea*, *Pedobacter* ↓ *Parabacteroides*, *Adlercreutzia*, *Collinsella*, *Lactobacillus*	/	[30]
		Adults	↑ Euryarchaeota, Verrucomicrobia, *Methanobrevibacter*, *Akkermansia* ↓ *Butyricimonas*, *Collinsella*, *Slackia*, *Prevotella*	/	[31]
ALS	Muscle weakness, atrophy, fasciculations, respiratory dysfunction	Adults	↑ *Bifidobacterium*	FMT	[32]
		Adults	↑ *Escherichia coli*, Enterobacteriaceae ↓ *C. baratii*, *C. hystoliticum*, *C. butyricum*, *C. prefringens*, *C. botulinum*, *C. tetan*	/	[33]
HD	Chorea, dystonia, cognitive decline	Adults	↓ Firmicutes, Verrucomicrobia, Akkermansiaceae, Lachnospiraceae, Acidaminococcaceae, Bacteroidaceae, Bifidobacteriaceae, Christensenellaceae, Clostridiaceae, Coriobacteriaceae, Enterobacteriaceae	/	[34]
		Mice	↑ Bacteroidales, Lactobacillales, Coriobacteriales, Erysipelotrichales, Bacteroidales, Burkholderiale ↓ Clostridiales, Clostridiales	*Bacillus subtilis* (Microecologics); FMT	[35]
		Mice	↑ Bacteroidetes, Actinobacteria, Proteobacteria ↓ Firmicutes, Deferribacteres	FMT	[36]
ADHD	Inattention, hyperactivity, impulsivity	Children	↑ Proteobacteria, *Shigella* ↓ Firmicutes, Bacteroidota	*Bifidobacterium bifidum* (Microecologics)	[37]
		Children	↑ *Bacteroides uniformis*, *Bacteroides ovatus*, *Sutterella stercoricanis*, Fusobacteria ↓ *Bacteroides coprocola*	/	[38]

Notes: FMT: fecal microbiota transplantation; 5AV: 5-aminovaleric acid; AD: Alzheimer’s disease; PD: Parkinson’s disease; MS: multiple sclerosis; ASD: autism spectrum disorder; ALS: amyotrophic lateral sclerosis; HD: Huntington’s disease; ADHD: attention deficit hyperactivity disorder. ↑: The bacterial population increases in the intestinal tract; ↓: The bacterial population decreases in the intestinal tract.

**Table 2:** Intervention effects of different types of microecologics on CNS diseases.

Types of Microecologics	Study Model	Doses	Intervention Duration	Results	References
**Probiotics**					
*Lactobacillus plantarum* PS128	ASD children	Take one capsule in total (3 × 10^10^ CFU/capsule)	4 weeks	Improved oppositional/defiant behavior	[132]
Probiotic combination (*Lactobacillus reuteri*, *Lactobacillus rhamnosus* and *Bifidobacterium infantis*)	AD mouse model	1 × 10^10^ CFU/d	10 weeks	Improved the spatial memory of rats, and reduced Aβ plaques and inflammation	[133]
LBS	MS adults	3.6 × 10^12^ CFU/d	2 months	Improved the structure of intestinal flora and inhibited the growth of harmful bacteria	[134]
*Lactobacillus casei* Shirota	PD adults	6.5 × 10^9^ CFU/d	6 weeks	Improved fecal consistency and reduced abdominal distension and pain	[135]
Probiotic capsules (*Lactobacillus acidophilus*, *Lactobacillus reuteri*, *Lactobacillus fermentum* and *Bifidobacterium*)	PD adults	8 × 10^9^ CFU/d	12 weeks	Improved cognitive function	[136]
SLAB51 (*Lactobacilli* and *Bifidobacterium*)	AD mouse model	2 × 10^11^ CFU/kg/d	4 months	Improved cognitive impairment	[137]
*Lactobacillus rhamnosus* GG ATCC53103	ADHD children	8 × 10^9^ CFU/d	3 months	Improved emotional, physical, social, and academic functioning	[138]
*Akkermansia muciniphila*	ALS mouse model	/	3 months	Alleviated the motor symptoms of ALS mice and prolonged their survival time	[139]
**Prebiotics**					
Lactulose	AD mouse model	200 mg/kg/d	4 weeks	Improved cognitive impairments	[140]
MOS	AD adults	(0.12% *w*/*v* in the drinking water, with a purity of 85%) replaced twice a week	8 weeks	Improved behavioral and cognitive disorders	[10]
Fructooligosaccharides from *Morinda officinalis*	AD mouse model	100 mg/kg/d	8 weeks	Improved learning and memory ability	[141]
B-GOS^®^	ASD children	/	6 weeks	Improved anti-social behavior	[142]
**Synbiotics**					
Synbiotic 2000 Forte (*Pediococcus pentoseceus* 5–33:3, *Leuconostoc mesenteroides* 32–77:1, *L. paracasei* ssp. *paracasei* 19, and *L. plantarum* 2362, as well as 2.5 g inulin, oat bran, pectin, and resistant starch)	ADHD children	1 × 10^10^ CFU/d	9 weeks	Improved emotion regulation ability	[138]
Novel synbiotic (PM + LGG)	PD adults	PM (30 mg/kg/d) + LGG (1.5 billion CFU/kg/d)	5 weeks	Promoted neuroprotection	[143]
**Postbiotics**					
Sodium butyrate	PD mouse model	200 mg/kg/d	3 weeks	Improved cognitive behavior and coordination ability	[144]
Sodium butyrate	ALS mouse model	2% sodium butyrate/d	2.5 months	Delayed disease onset and prolonged the lifespan of ALS mice	[145]

Note: CFU: colony-forming unit; kg: unit of body weight; LBS: multi-strain probiotics (*Lactobacillus paracasei* DSM 24734, *L. plantarum* DSM 24730, *L. acidophilus* DSM 24735, *L. delbruckei* subspecies *bulgaricus* DSM 24734, *Bifidobacterium longum* DSM 24736, *B. infantis* DSM 24737, *B. breve* DSM 24732, *Streptococcus thermophilus* DSM 24731); MOS: mannan oligosaccharide; B-GOS^®^:Bimuno^®^ galactooligosaccharide; PM: Polymannuronic acid; LGG: *Lacticaseibacillus rhamnosus* GG; AD: Alzheimer’s disease; PD: Parkinson’s disease; MS: multiple sclerosis; ASD: autism spectrum disorder; ALS: amyotrophic lateral sclerosis; ADHD: attention deficit hyperactivity disorder.

## Abbreviations

GM: gut microbiota; CNS, central nervous system; MGBA, microbiota–gut–brain axis; AD, Alzheimer’s disease; PD, Parkinson’s disease; MS, multiple sclerosis; ASD, autism spectrum disorder; ALS, amyotrophic lateral sclerosis; HD, Huntington’s disease; ADHD, attention deficit hyperactivity disorder; Aβ, amyloid-beta; APP, amyloid precursor protein; GF, germ-free; SPF, specific pathogen-free; FMT, fecal microbiota transplantation; ENS, enteric nervous system; ASO, α-synuclein-overexpressing; MPTP, 1-methyl-4-phenyl-1,2,3,6-tetrahydropyridine; NDD, neurodevelopmental disorder; GI, gastrointestinal; LPS, lipopolysaccharide; SCFAs, short-chain fatty acids; EAE, experimental autoimmune encephalomyelitis; SFB, segmented filamentous bacteria; TMAO, trimethylamine *N*-oxide; BBB, blood–brain barrier; GABA, gamma-aminobutyric acid; GLP-1, glucagon-like peptide 1; HN, hypothalamic–neurohypophyseal; HPA, hypothalamic–pituitary–adrenal; BDNF, brain-derived neurotrophic factor; GBA, gut–brain axis; DCs, dendritic cells; HDACs, histone deacetylases; TNF, tumor necrosis factor; 4-EPS, 4-ethylphenylsulfate; GPCRs, G protein-coupled receptors; FFARs, free fatty acid receptors; TLRs, Toll-like receptors; PSA, polysaccharide A; 5-AVA, 5-aminovaleric acid; WHO, World Health Organization; FOS, fructo-oligosaccharide; GOS, galacto-oligosaccharide; 5-HT, 5-hydroxytryptamine; 5-HIAA, 5-hydroxyindoleacetic acid; B-GOS, bimuno-galacto-oligosaccharide; ISAPP, International Scientific Association for Probiotics and Prebiotics; NMN, nicotinamide mononucleotide; IL-1β, interleukin 1β; IL-6, interleukin 6; TNF-α, tumor necrosis factor alpha; ROS, reactive oxygen species; PPAR-γ, peroxisome proliferator-activated receptor gamma; EPS, exopolysaccharide; DA, dopamine; NFTs, neurofibrillary tangles; IECs, intestinal epithelial cells; FMO3, flavin-containing monooxygenase 3; IL-10, interleukin 10; IgA, immunoglobulin A; AhR, aryl hydrocarbon receptor; Th17, T helper cell 17; ACh, acetylcholine; SGB, species-level genomic bin; EECs, enteroendocrine cells; DM, diabetic mice; ABCA1, ATP-binding cassette transporter A1; apoA-I, apolipoprotein A-I; HDL, high-density lipoprotein; LPC, lysophosphatidylcholine; CPZ, cuprizone; ETC, electron transport chain; GPx, glutathione peroxidase; GSH, glutathione; NE, norepinephrine; BCAAs, branched-chain amino acids; LAT1, L-type amino acid transporter 1; p-AKT, phosphorylated protein kinase B; p-PKC, phosphorylated protein kinase C; p-TrkB, phosphorylated tyrosine kinase receptor B; 6-OHDA, 6-hydroxydopamine; TrkB, tropomyosin receptor kinase B; AMPK, AMP-activated protein kinase; PGC-1α, peroxisome proliferator-activated receptor γ coactivator 1α; SE, endurance; ARE, antioxidant response element; MD, Mediterranean diet; MDS-UPDRS, Movement Disorder Society Unified Parkinson’s Disease Rating Scale; OXT, oxytocin; AVP; arginine vasopressin.
